# Learning Document Semantic Representation with Hybrid Deep Belief Network

**DOI:** 10.1155/2015/650527

**Published:** 2015-03-23

**Authors:** Yan Yan, Xu-Cheng Yin, Sujian Li, Mingyuan Yang, Hong-Wei Hao

**Affiliations:** ^1^Department of Computer Science and Technology, School of Computer and Communication Engineering, University of Science and Technology Beijing, Beijing 100083, China; ^2^Key Laboratory of Computational Linguistics, Peking University, Ministry of Education, Beijing 100871, China; ^3^Institute of Automation, Chinese Academy of Sciences, Beijing 100190, China

## Abstract

High-level abstraction, for example, semantic representation, is vital for document classification and retrieval. However, how to learn document semantic representation is still a topic open for discussion in information retrieval and natural language processing. In this paper, we propose a new Hybrid Deep Belief Network (HDBN) which uses Deep Boltzmann Machine (DBM) on the lower layers together with Deep Belief Network (DBN) on the upper layers. The advantage of DBM is that it employs undirected connection when training weight parameters which can be used to sample the states of nodes on each layer more successfully and it is also an effective way to remove noise from the different document representation type; the DBN can enhance extract abstract of the document in depth, making the model learn sufficient semantic representation. At the same time, we explore different input strategies for semantic distributed representation. Experimental results show that our model using the word embedding instead of single word has better performance.

## 1. Introduction

Semantic representation [1–3] is very important in document classification and document retrieval tasks. Currently the main representation method is bag-of-words, but this method only contains the word frequency information, which is very shallow, and this representation is not enough. Therefore, many researchers began to explore deeper representations. LSI [4] and pLSI [5] are two kinds of dimension reduction methods which use SVD (Singular Value Decomposition) to operate on a document vector matrix and remap it in a smaller semantic space than the original one. But this method can still only capture very limited relations between words. Blei et al. [6] proposed Latent Dirichlet Allocation (LDA) that can extract some document topics which has shown superior performance over LSI and pLSI. This method is popular in the field of topic model; in the meantime, it is also considered a great method for reducing dimensions. But this method has some disadvantages: semantic features of the study are not sufficient for the documents, exact inferences in the directed model are intractable [7, 8], and it cannot properly deal with documents of different lengths.

More recently, deep learning [9] has evolved as a new branch of the research field of machine learning. This method greatly enhances semantic representation of the document. Some researchers have started the work; for example, Hinton and Salakhutdinov proposed a two-layer undirected graphical model [10] called “Replicated Softmax model” (RSM) to explore the use of basic deep learning methods to represent the document information, which had a better result than LDA method; Larochelle proposed “Doc Neural Autoregressive Distribution Estimation” (DocNADE) that was inspired by RSM and similar to an autoencoder neural network [8, 11]. In this model the input vector of observations has the same size as the output vector. This method showed that the DocNADE is competitive not only as a generative model of document but also as a learning algorithm for extracting meaningful representation of documents. The high-level abstractions through these deep network models achieve higher generalization than probabilistic topic models in terms of unseen data [12]. However these methods use the weight-sharing method and only have two layers, which is not enough to sufficiently learn about deeper representation. Because the document is missing large quantity of information in the dimension reduction process, the high level of models for different documents indicates there is little difference of learning, which did not lead to very good result in document classification and retrieval. In document classification and retrieval tasks, the most important and only used factor is the high-level document vector distributed representation of the model, so we must find a vector that can represent most messages that indicate the current document.

Based on the above disadvantage, in this paper, we propose a new model called Hybrid Deep Belief Network (HDBN) which is improved by the DBN (Deep Belief Network) [13]. First, we use two-layer DBM (Deep Boltzmann Machine) [14] which can be utilized to reduce the dimension and remove noise in the lower layer of the HDBN model to extract abstract of the document, and preserving the most of the documents' information, which is an effective way to improve performance; secondly, we use the DBN model reduction effect again to obtain the deeper document representation in the output nodes dimension.

Then, we perform a variety of experimental studies for learning document semantic representation with HDBN. In the first part of our experiments, we compare our HDBN model with the RSM, DocNADE, and regular DBN model, and the experiment result shows that our HDBN model has a better result for document classification and retrieval in two datasets. In order to get an even better semantic representation of a document based on deep learning, we also explore the effects of different inputs on the model. In the second part of our experiments, we conduct several experiments about semantic representation, and the experiment results are elaborated in Section 4.3.

## 2. Hybrid Deep Belief Network

Deep Learning is based on distributed representations, and different numbers and sizes of layers can be used to provide different amounts of abstraction. The higher level is developed from the lower level, and these models are often composed of a greedy layer-by-layer method. The whole model includes the pretraining and fine-tuning processes, which are helpful to explore the high-level abstraction. In this section, we first describe the notable deep learning method, Deep Belief Network (DBN), and Deep Boltzmann Machine (DBM). Then we introduce our improved deep learning model, HDBN, and its training method.

### 2.1. Deep Belief Network

Hinton and Salakhutdinov [9] introduced a moderately fast, unsupervised learning algorithm for deep models called Deep Belief Networks (DBN). The DBN can be viewed as a composition of stacked Restricted Boltzmann Machines (RBMs) that contain visible units and hidden units. The visible units represent the document data and the hidden units represent features learned from the visible units. Restricted Boltzmann Machine [15] is a generative neural network that can learn probability distribution over its set of inputs. An RBM is a kind of Boltzmann Machine in which all the visible units are connected with hidden units while having no connection within visible layer.

Each RBM layer can capture high correlations of hidden features between itself and the layer below. An RBM can be used as a feature extractor. After successful learning, an RBM gets a closed-form representation from the training data. In the training process, Gibbs samples are useful to obtain an estimator of the log-likelihood gradient. An RBM is composed of both visible units and hidden units. When a visible unit *x* is clamped to the observed input vector, first we can get a hidden unit *h* from *x* and then get a new visible unit *x*′ from unit *h* by the Gibbs sampling. Although when using the Gibbs sampling we can get the log-likelihood function on the unknown parameters of the gradient approximation, typically it takes a larger number of steps in the sampling, which makes the efficiency of RBM training low, especially when we have the observation data with high-dimensions. Hinton proposed the idea of *k*-step Contrastive Divergence (CD-*k*) which has become a fast algorithm for training RBM [12, 13]. The surprising empirical result is that even when *k* = 1 (CD-1), it still can get good results. Contrastive Divergence has been used as a successful update rule to approximate the log-likelihood gradient in training RBMs [16, 17]. Through this Contrastive Divergence algorithm, we can improve the efficiency of the model training.

### 2.2. Deep Boltzmann Machine

Deep Boltzmann Machine [14, 18, 19] is a network of symmetrically coupled stochastic binary units, and it is also composed of RBMs. It contains a set of visible units and hidden units. Unlike DBN model, all connections between layers in the DBM model are undirected. DBM has many advantages: it retains and discovers layers presentation of the input with an efficient pertaining procedure; it can be trained on unlabeled data and parameters of all layers can be optimized jointly in the likelihood function. However, DBM has a disadvantage that the training time grows exponentially with the machine's size, and the number of connection layers, which makes large-scale learning of DBM model uneasy. So we just reduce the document dimension and remove noise with DBM model in the lower layers and then continue training with DBN model, which guarantees that the document can have a good feature extraction and reduces training time at the same time when we need some layers in the model.

### 2.3. HDBN Model

#### 2.3.1. Principle Analysis

DBM composed of two-layer RBMs can learn better representation because when training parameters, each state of the hidden layer node is determined by the lower and higher level together which directly connected to the layer, and this is the model's characteristic which is undirected graph model and motivation of our models using DBM training. Besides, we analyze the data structure of the documents and find that using DBM can remove noise brought by the document input. But judging from the combined effect, the effect of DBM with more than two layers is not as good as that of DBN model with more than two layers. One reason is that when using DBN to train, the parameters are prone to be overfitting; another reason is that the DBM training has much higher complexity than the DBN training, and its training time is three times that of DBN [14].

**Figure 1:**
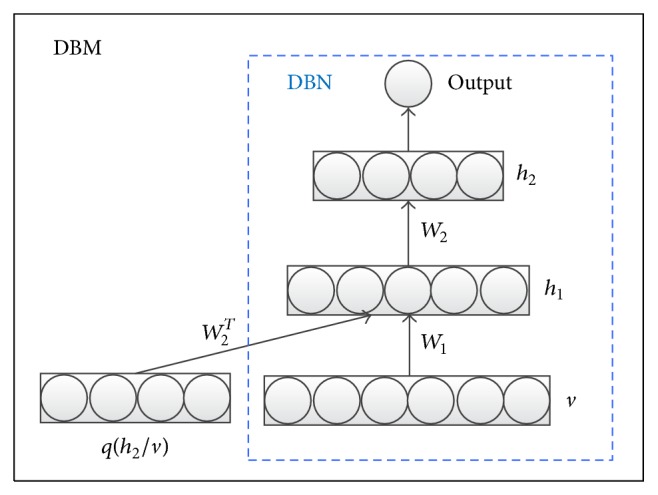
Hidden layer nodes construction of DBN and DBM.

Summarily, the directed graph model (DBN) has some limitations, since it only uses visible nodes from previous layer. Therefore, the DBM model proposed by Salakhutdinov and Hinton [19] uses the undirected graph model. DBM uses both the former layer and the next layer's nodes, and the hidden nodes sampling will be more accurate.  Figure 1 shows the construction and learning of hidden layer nodes in DBM and DBN, where *q*(*h*_2_∣*v*) is an approximate posterior distribution. For DBN, nodes are constructed as in the way in directed graphs and only depend on their previous layer. However, for DBM, nodes are constructed and learned as in the way in undirected graphs. We hope our proposed hybrid model (HDBN) can combine several advantages from both directed and undirected graph models.

#### 2.3.2. Framework Analysis

**Figure 2:**
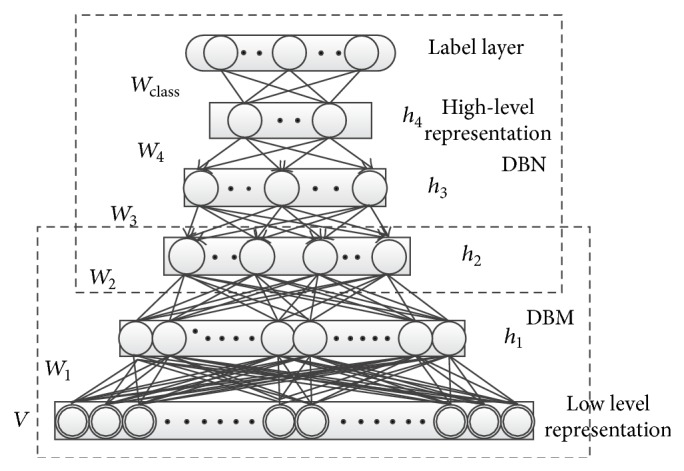
Hybrid Deep Belief Network.

Considering the limitations of DBN and DBM, especially for document representation, in this paper, we propose HDBN (see Figure 2) which uses the Deep Boltzmann Machines model composed of simple two-layer RBMs in the lower layers and Deep Belief Networks model made up of two-layer RBMs in the upper layers as we take both training time and the model accuracy into consideration for document classification and retrieval tasks. Our HDBN model has four layers. *v* is the visual layer which is also the inputs of model, and each document is represented by a fixed length vector. *h*_1_, *h*_2_, *h*_3_, and *h*_4_ are four hidden layers with different number of nodes for each level. Nodes with the same hidden layer are not connected with each other; for nodes of different layers, between hidden layer *h*_1_ and *v* as well as hidden layer *h*_2_, there are undirected fully connections and between *h*_3_ and *h*_4_ as well as *h*_2_ there are directed connections. After the document is represented as a fixed vector, it will be trained by the first two layers *h*_1_ and *h*_2_ of DBM training, and *h*_2_ is the output of DBM; at same time, *h*_2_ is the input of the DBN model which is made of *h*_3_ and *h*_4_, and *h*_4_ is the final output of the HDBN model. Compared with the visual layer, we call it high-level semantic representation. Document classification and retrieval tasks are based on this output vector.

In order to get the optimal parameters for the model, Hinton and others introduce a model of energy where whether one can get optimal solutions embedded in the energy function is critical. One of the major works of statistical pattern recognition is to capture correlations between variables, and it is the same as energy model. RBM energy function [13, 20] is

(1) $$\begin{array}{c} E\left(v,h\right)=-\overset{n}{\underset{i=1}{\sum }}\overset{m}{\underset{j=1}{\sum }}W_{ij}h_{i}v_{j}-\overset{m}{\underset{j=1}{\sum }}b_{j}v_{j}-\overset{n}{\underset{i=1}{\sum }}c_{i}h_{i} \end{array}$$

in which *n* is the number of hidden nodes, *m* is the number of visible nodes, and *b* and *c* are the biases of visual layer and hidden layers, respectively. This formula is the energy function which represents each of the connections between visible and hidden nodes. The objective function of RBM is the cumulative value of energies of all visible and hidden nodes, but, as for the objective function, in each sample we need to know values of all the hidden nodes that it can correspond to in order to calculate energy, so we will face index level of difficulty in calculation. The solution is to switch the energy problem to the problem of the probability of the model. The joint probability [13] of visible and hidden nodes is

(2) $$\begin{array}{c} P\left(v,h\right)=\frac{e^{-E\left(v,h\right)}}{\sum_{v,h}^{}e^{-E\left(v,h\right)}}. \end{array}$$

By introducing this probability we can easily solve energy model, and our goal is to get the minimum energy. In statistical mechanics, the lower energy state has a higher probability of occurrence than the higher energy state, so we want to maximize the probability. The introduction of free energy function [13, 20] is

(3) $$\begin{array}{c} \text{Free}\;\text{Energy}=-\ln\underset{h}{\sum }e^{-E(v,h)} \end{array}$$

so we have

(4) $$\begin{array}{c} P\left(v\right)=\frac{e^{-\text{Free}\;\text{Energy}\left(v\right)}}{Z},\;\;Z=\underset{v,h}{\sum }e^{-E\left(v,h\right)}, \end{array}$$

in which *Z* is the normalization factor. Consider

(5) $$\begin{array}{c} \ln P\left(v\right)=-\text{Free}\;\text{Energy}\left(v\right)-\ln Z \end{array}$$

and in this formula the first item on the right is the negative values of the comprehensively total free energy function of the entire network; on the left it is the likelihood function, so this is just as what we have said in introduction to model, using the maximum likelihood estimate for solving model parameters.

## 3. Feature Representation

The benefits of the deep learning approaches are that they can flexibly use various features and automatically learn some latent information. First we just use the simplest and most popular approach to represent a document, that is, the use of bag-of-words (BoW) as the visible units. With this kind of features, we compare HDBN with the other existing methods. In the field of Natural Language Processing (NLP), if we know additional complex features (e.g., word embedding), then we may get more precise semantic representation to implement the document classification task more efficiently. Thus, in our work, we explore whether using the word embedding [21–23] as the visible input will have advantages over using the BoW as input for both the document classification and document retrieval tasks.

### 3.1. High-Dimensional Word Embedding Feature Representation

**Figure 3:**
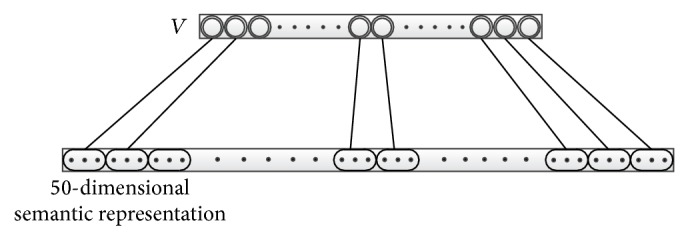
The 50-dimensional word embedding.

The input vector of BoW only includes the word frequency information of documents, which also means that in the vector every word is represented in one-dimensional element regardless of their different contribution to the document. As we know, different words contribute to the understanding of a document to different extent and a single numerical value is difficult to represent a word well. Thus, it is better to explore the semantic representation of each word. Huang et al. [24] trained with a 50-dimensional word embedding to represent every word by using both local and global context information, and these representations can capture the semantic and syntactic information of words. The results of evaluation on the word similarity showed that the words from more similar categories or with similar properties have closer distance in multidimensional space. Thus we want to explore whether the 50-dimensional word embedding can help extract deeper semantic representation. However, from Figure 3, we can see that this input vector will be much longer than the original BoW type, so we call it a high-dimensional semantic representation. We give detailed descriptions in the experimental section.

### 3.2. Keyword-Based Low-Dimensional Word Embedding Feature Representation

Apart from this high-dimensional vector representation, we also try inputting a low-dimensional representation in contrast with BoW to see whether to achieve better results. During our training using the HDBN model, we found that when the dimension of input vector is small, the training time decreases significantly. So we want to select some keywords of the document using term frequency-inverse document frequency (TF-IDF) [25], and in order to achieve a better result we used the document label when we calculate the IDF. We use that

(6) $$\begin{array}{c} \text{IDF}=\log\frac{N*n}{m+k} \end{array}$$

and in this formula *N* is the total number of documents, *n* is the number of documents containing the word in current category, *m* is the total number of documents in current category, and *k* is the number of documents containing the word in other categories. This input can reduce the number of visible units and ensure semantic information of the document at the same time, which results in fast training and high precision. We calculated the TF-IDF of each word in the document and sorted the words according to their TF-IDF values and then took the top 40 words as the keywords. In contrast with the high-dimensional representation, we call it keyword-based low-dimensional representation.

## 4. Experiments

In this section, we conducted two sets of experiments. In the first set of experiments, we only observed which model has better semantic representation from BoW input. In the second set of experiments, we compared high-dimensional vector and keyword-based low-dimensional vector with bag-of-words to find out which one is the most suitable semantic distributed representation for our HDBN model applied to document classification and document retrieval tasks.

In our experiments, two datasets are used: 20 Newsgroups and BBC News data. They are all very popular in document classification and document retrieval. The 20 Newsgroups dataset is also used in the RSM model paper [10], so we can compare the two different methods effectively.

### 4.1. Description of Datasets

*20 Newsgroups Data*. The data is organized into 20 different newsgroups, each corresponding to a different topic. The website has three versions, and we selected the third one with 18828 documents. This version does not include cross posts and includes only the “From” and “Subject” headers. We randomly divided the data into training part and test part. In our experiment we selected 11340 documents as the training set and 7478 as the test set. After removing stop-words and stemming, we consider the 2000 as the most frequent words in the training dataset.

*BBC News Data*. The data is made up of news articles from the BBC. BBC data contains 2225 documents corresponding to five topical areas (business, entertainment, politics, sport, and tech). In experiment we selected 1569 documents as the training set and 656 as the test set. As with the 20 Newsgroups data, we process the BBC data in the same way.

### 4.2. Details of Training

In the whole experiments, since there are many samples in our dataset, in order to improve effectiveness of the training, we divided the training samples into several batches, and every batch has 100 examples. In contrast to our HDBN with regular DBN, we chose four levels in regular DBN model and each RBM was trained by 50 iterations in the pretraining process, and afterwards, back propagation was used for overall fine tuning in the two models. We chose 0.01 as the weight update parameter of the formula in the HDBN, DocNADE, and regular DBN model. However, in the first experiment which compared the RSM, DocNADE, and regular DBN model with our HDBN, we do not know the author's weight parameters in RSM, so we did experiments with three different parameters 0.01, 0.001, and 0.0001 and selected per-batch instead of per-epoch when we calculated reconstruction error. The high-level distributed representation learned through these models can be seen as latent words of the document, so the number of latent words will have an impact on document classification and retrieval. In this experiment, we set the numbers of output nodes as 50, 100, 128, 500, and 1000.

In the second experiment, we were able to use Huang's training corpus dictionary and it had been public. As we described in Section 3.1, every word is replaced by the 50-dimensional word embedding, so in high-dimensional representation experiment, the visible units will become 100,000-dimensional. In the 2000-most-frequent-words vector every element contains the corresponding word frequency, so after processing the 100,000-dimension vector we also needed to set a weight to each element. This weight was computed by the word frequency and the dictionary word TF-IDF. As we have described in Section 3.2, in the keywords-based low-dimensional representation experiment, we used TF-IDF method to select 40 keywords; then we continued using the 50-dimensional word embedding to replace the keywords as the input vector.

### 4.3. Experimental Results

We selected document classification and retrieval tasks to evaluate experiments. Document classification assigns a document to one or more classes. In our experiments, every document is assigned to only one class. Our evaluation criteria is that if the class of current test document we get from the model is same as its actual class, then we think the result is correct. We chose softmax function in these three models. Document retrieval is used to decide whether a retrieved document is relevant to the query document by simply checking if they have the same class label. For the Replicated Softmax and Deep Belief Network, we also utilized the high-level distributed representation vector to calculate similarity with the cosine of the angle.

#### 4.3.1. Experiment with Different Models

In our models, all the inputs are 2000-dimensional, and the output vector is high-level semantic representation of the document.  Table 1 shows the accuracy of document classification and the precision result of document retrieval. In order to contrast HDBN with RSM and DocNADE, the number of highest layer units in HDBN model needs to be the same as in RSM, as described in Section 4.2. In the HDBN model, we chose different numbers of nodes for different layers. For example, in Table 1, when the output number is 50, the nodes in these layers are set up to 2000 (input), 1500, 800, 400, 50 (output) respectively. The output unit number refers to the number of output nodes in the model. L_r is the learning rate we use for training the model as we have described in Section 4.2. From the two datasets we found that our HDBN model is superior to RSM, DocNADE, and regular DBN model. In addition, our HDBN model also has a high precision in document retrieval compared with Over-Replicated Softmax model which is improved by RSM [7]. This result is because not only HDBN has more layers, which is a main advantage of deep learning to learn samples more sufficient, but also the model can adjust the weights that connect the nodes between visible and hidden neurons which are more flexible.

In order to find more reasons why the HDBN can retrieve more similar document than RSM model can we found that even though the same test document has the same retrieval result from the train samples through the two models, the calculated cosine values are different. We found that the cosine value calculated in the RSM model is bigger than that in the HDBN model. The input vector of the document is the same but the sample's outputs from the two models are different, and the bigger cosine value means the two samples are more similar. The HDBN model's cosine is smaller meaning that the test document representation from the model output nodes dimension is more refined. This also means the HDBN model can learn deeper distributed representation from documents between different samples which can help the new test document retrieve more similar sample from the train samples.

DocNADE is a generative model inspired by RSM. They both use shared weights, which mean different visible nodes that are connected to the same hidden layer have the same weight. The difference is that all RSM's hidden layers are connected with visible nodes, while DocNADE's hidden nodes are not. They use fully visible Bayesian networks, which decompose the observation's probability distribution. After training by DocNADE, we will have more valuable information to identify intruder words in the documents [8]. With these intruder words, we can do better when predicting topics compared with RSM, which use whole documents for prediction. However, for the redundant datasets, intruder words will not be effective. Although shared weights can reduce the time for parameters training and fine-tuning, our HDBN with directed and undirected connections can significantly reduce sampling errors.

We can also see from Table 1 that HDBN model has better performance than regular DBN model. With the same number of levels in two models (both have four levels), the difference is that using DBM training instead of DBN training in the previous two-layers. This undirected graph model can adjust the parameters of the model to be more flexible; we can make the upper DBN a better display of abstract function in training.

Even though we constantly optimize the model, the 20 Newsgroups dataset still has low accuracy of classification and retrieval results. So we analyze the results from the experiments and find in the 20 Newsgroups dataset that the same document appears in different categories which means the dataset contains some wrong information. However, inspired by that, we think there is a way to increase the robustness of the model. In addition to the wrong information of the dataset, we also find that some categories are very similar to other categories, for example, the category alt.atheism is very similar to the soc.religion.christian and talk.religion.misc category which increase the difficulty in our classification. In order to prove our model, we also do the document classification and retrieval experiments with six categories in the 20 Newsgroups. The six categories are also divided on the dataset website. From the six categories experiment on the 20 Newsgroups we can see that the model can get a good classification and retrieval result.  Table 2 shows the accuracy of document classification and the precision result of document retrieval on the six categories about the 20 Newsgroups.

#### 4.3.2. Experiment with Different Inputs

After the high-dimensional (100,000) experiment, we found that the experiment result is not satisfactory. The model's result only gives out the same category for different samples. We checked the different layer's representation and tried to find the reasons. We found that in the back propagation process the classification accuracy does not improve even though the epoch increases, and each specific layer of units representation from different samples is the same; that is, there is no difference among samples. So we assume the weights of the model may not fit well in learning. But when we rechecked the pretraining process, we found that the weights in every RBM which connected the hidden units with visible units are different from each other and the reconstruction error is also very small; however, every layer of hidden units representation becomes the same after the neuronal activation function, and that is why the highest layer has the same abstraction in every sample which led to the unsatisfactory result of our model. Our analysis of the results indicates two reasons: the first is that the layer number of HDBN model is small. The four layers are not enough for visible layer which has 100,000 units, because it will lose too much information. The second reason is that the 100,000 inputs are too sparse to lead the model to do good work. Because the average length of each document is about 100 words, even though the percentage of nonzero elements in the 100,000 vector is same as in the 2000 vector, there are still about 95,000 zero elements which make the vector too sparse.

To address the first reason, we tried to increase number of layers to 8, but we still got the same result which is that each RBM weights are different while each hidden unit representation is the same. In order to solve the problem of sparse data, we deal with the data through normalization processing. After the processing, there is still no significant change of the experiment result because the zero elements are only replaced by other repeating elements after normalization.

Even though our experiment result does not seem good enough, we think that the 50-dimensional representation for every word is still helpful semantic representation, so we want to explore whether this words' semantic representation can play an important role if we reduce the dimension of the input. To be more specific, we used some keywords instead of bag-of-words and every word is also represented by a 50-dimensional vector.

In the keyword-based short-dimensional experiment, the results (see Table 3) show that the method of combining extract document keywords with word embedding is more helpful for HDBN model to extract semantic information compared with BoW. From the results we analyze the reason why keyword semantic input can get more accuracy than BoW input. We found two semantic representations from the keyword word embedding method. From the input of the document, we found that the different documents in the same category using the TF-IDF keyword representation have more difference than using the BoW representation. This is the first semantic selection. After this, the representation can filter out high frequency words in the same category and keep a few low frequency words in the same category which appear in other documents. This representation can help the test document find the similar and relevant document because the selected keywords are more representative. The second semantic representation is the 50-word embedding representation. This representation makes the similar documents closer to each other in the space of model output nodes dimension. Not just semantic representation, we also found in the experiment that using DBM training in the preview layers, except for the flexible adjustment of parameters we have analyzed in Section 2.3, the keyword word embedding input will bring an unnecessary noise while having the benefit of keywords and training with HDBN can get rid of the noise for the model. It also provides a direction for our next research work, which is, trying to change the input type.

**Figure 4:**
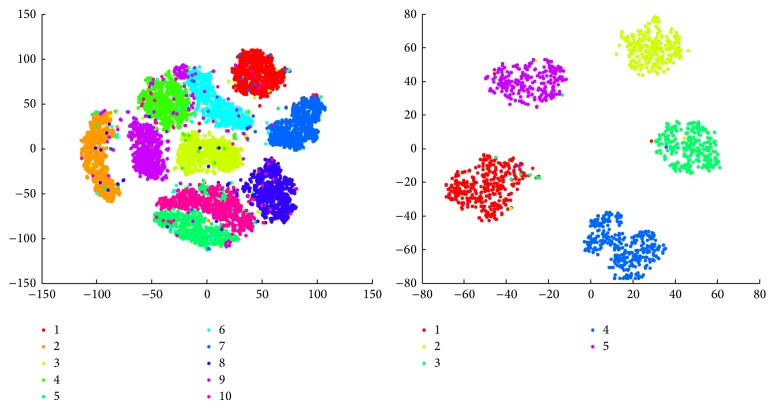
20 Newsgroups and BBC documents representation visualization.

We also do visualization using the outputs from the model and we find that the semantic distributed representation is more concentrated using the keyword word embedding input than using the BoW input.  Figure 4 shows visualization results of the document distributed representation. Due to the limited space here we only randomly choose 10 categories of 20 Newsgroups for visualization. The points with different colors, respectively, indicate the different classes of the corresponding documents. We use t-SNE [26] to visualize our results. The scales of axes are automatically generated based on the number of datasets. Here, we can see that the short word embedding representation of TF-IDF keywords has the same representation role like that of BoW (with 2000 dimension), since the word embedding representation contains more semantic information.

## 5. Conclusion

We explored the effects of different input on our HDBN model for extracting semantic information. In HDBN model, we used the advantage of DBM model to extract representation and remove noise in the lower layers which is different from regular DBN and then used CD sampling to speed up the training process. The experiment results of document classification and retrieval on two datasets show that our improved HDBN model is much better than other models. We used 50-dimensional word embedding learning from the dictionary of Huang instead of each word in bag-of-words, and the experiment results show that the semantic word embedding is more helpful for HDBN model to extract semantic information. TF-IDF is a simple and classic method for extracting keywords from documents, but it failed to reflect the location of the word in the document, especially in the case of Web documents where calculation of the weighting method should reflect the structure of HTML features. In the future we are going to explore the use of other keyword extraction methods, for example, with Wikipedia.

## Figures and Tables

**Table 1:** Document classification accuracy and document retrieval precision.

Dataset	Model	L_r	Document classification					Document retrieval				
			Output units number					Output units number				
			50	100	128	512	1000	50	100	128	512	1000
20 Newsgroups	RSM	0.01	63.11	66.82	67.67	66.52	69.31	62.82	67.80	68.64	65.10	67.48
		0.001	60.94	62.91	71.55	73.68	73.05	56.10	58.32	69.17	69.65	69.34
		0.0001	36.59	62.75	63.33	70.40	71.38	28.46	58.56	59.39	66.37	65.14
	DBN	0.01	72.77	72.35	72.49	72.56	72.39	67.52	67.78	68.90	69.27	69.81
	DocNADE	0.01	73.83	73.40	73.54	74.75	74.11	68.50	68.78	70.17	70.66	70.82
	HDBN	0.01	**76.57**	**76.68**	**76.35**	**76.49**	**76.89**	**76.91**	**71.89**	**72.99**	**73.50**	**73.91**

BBC data	RSM	0.01	94.04	94.65	95.41	95.11	95.87	93.44	93.29	92.98	93.90	94.81
		0.001	95.11	96.48	96.64	94.65	96.64	94.66	95.34	96.18	95.79	96.03
		0.0001	60.40	94.65	94.80	96.79	94.95	70.88	91.76	94.05	95.88	95.42
	DBN	0.01	95.31	94.81	96.03	95.06	95.05	93.18	94.10	94.30	94.13	94.69
	DocNADE	0.01	95.60	96.77	96.93	96.89	96.93	94.94	95.44	96.28	95.98	96.13
	HDBN	0.01	**97.40**	**97.40**	**98.01**	**97.09**	**97.25**	**95.42**	**96.02**	**96.94**	**96.48**	**96.94**

**Table 2:** Document classification accuracy and document retrieval precision of six categories on 20 Newsgroups.

Dataset	Model	L_r	Document classification					Document retrieval				
			Output units number					Output units number				
			50	100	128	512	1000	50	100	128	512	1000
20 Newsgroups	RSM	0.01	76.13	79.60	80.63	80.24	80.61	75.40	79.83	80.24	78.64	79.66
		0.001	74.11	76.20	81.88	81.03	81.34	68.23	70.43	80.89	81.08	81.07
		0.0001	57.88	76.89	76.92	83.34	82.93	58.66	71.98	72.64	80.45	78.93
	DBN	0.01	76.52	79.77	81.03	82.94	82.11	77.36	80.21	80.07	80.99	79.01
	DocNADE	0.01	78.82	81.37	82.70	83.67	83.76	78.13	81.01	81.46	81.81	81.15
	HDBN	0.01	**85.92**	**85.77**	**86.82**	**86.94**	**86.15**	**82.32**	**82.42**	**84.97**	**84.87**	**83.73**

**Table 3:** Document classification accuracy and retrieval precision using keyword word embedding.

Dataset	Document classification					Document retrieval				
	Output units number					Output units number				
	50	100	128	512	1000	50	100	128	512	1000
20 Newsgroups	81.57	81.68	82.35	82.09	82.29	81.91	76.89	78.99	78.50	78.91
BBC data	98.41	98.84	99.35	98.82	97.76	97.26	96.97	98.05	97.52	98.58
